# Molecular phylogeny and morphology reveal two new species and one new record of *Ophiocordyceps* (*Ophiocordycipitaceae*, *Hypocreales*) parasitic on *Hymenoptera* in China

**DOI:** 10.3897/mycokeys.137.191268

**Published:** 2026-07-29

**Authors:** Ze-Mei Chen, He-Fa Liao, Fang-Wei Lou, Rui Hu, Jin-Long Jia, Wen-Bo Zeng, Zhu-Liang Yang, Ai-Li Zhang, Yuan-Bing Wang

**Affiliations:** 1 School of Chinese Materia Medica, Yunnan University of Traditional Chinese Medicine, Kunming 650500, Yunnan Province, China State Key Laboratory of Phytochemistry and Natural Medicines, Kunming Institute of Botany, Chinese Academy of Sciences Kunming China; 2 State Key Laboratory of Phytochemistry and Natural Medicines, Kunming Institute of Botany, Chinese Academy of Sciences, Kunming 650201, China Yunnan Key Laboratory for Fungal Diversity and Green Development, Kunming Institute of Botany, Chinese Academy of Sciences Kunming China; 3 Yunnan Key Laboratory for Fungal Diversity and Green Development, Kunming Institute of Botany, Chinese Academy of Sciences, Kunming 650201, China School of Chinese Materia Medica, Yunnan University of Traditional Chinese Medicine Kunming China; 4 Sanqi Medicines College, Wenshan University, Wenshan 663099, China Sanqi Medicines College, Wenshan University Wenshan China

**Keywords:** *
Hymenostilbe
*, morphology, new species, phylogenetic analyses, taxonomy

## Abstract

Two novel species, *Ophiocordyceps yuanyangensis* and *O. gotrae*, along with a new record, *O. cylindrospora*, parasitic on hymenopteran hosts, were collected in Yunnan, China. Morphological observations and multi-gene (nrSSU, nrLSU, *tef*-1α, *rpb*1, and *rpb*2) phylogenetic analyses confirmed their placement in the *Hymenostilbe* clade within the genus *Ophiocordyceps*. When compared with closely related species, *O. gotrae* is distinguished by a smaller fertile head and shorter asci, whereas *O. yuanyangensis* shows smaller fertile parts and asci but larger perithecia and part-spores. Phylogenetic evidence supports their recognition as distinct taxa. To date, 24 species in the *Hymenostilbe* clade have been reported parasitizing *Hymenoptera*. This study enriches the species diversity of the *Hymenostilbe* clade and highlights the importance of integrating morphological and molecular data for accurate taxonomic identification.

## Introduction

The genus *Ophiocordyceps* Petch was established in 1931 to accommodate a distinct group of species originally classified under *Cordyceps* Fr. sensu lato, including *O. blattae* Petch, *O. unilateralis* (Tul. & C. Tul.) Petch, *O. peltata* (Wakef.) Petch, and *O. rhizoidea* (Höhn.) Petch (Petch 1931). *Ophiocordyceps blattae* was designated as the type species of *Ophiocordyceps*, characterized by asci with conspicuous apical caps and ascospores with conspicuous septation (Petch 1931, 1933). Throughout the 20^th^ century, however, this genus was subsequently treated as a subgenus of *Cordyceps* or placed in several subgenera of *Cordyceps* (Kobayasi 1941; Mains 1958). Its status as a separate genus was not formally reinstated until 2007, when Sung et al. revived *Ophiocordyceps* based on multi-gene phylogenetic evidence and established the family *Ophiocordycipitaceae* (Sung et al. 2007a).

To date, approximately 420 species of *Ophiocordyceps* have been accepted (https://www.mycobank.org; Sung et al. 2007a; Spatafora et al. 2015; Araújo et al. 2018; Luangsa-ard et al. 2018; Tang et al. 2022; Tang et al. 2023; Mongkolsamrit et al. 2024; Liao et al. 2025). Members of this genus are typically characterized by fibrous, rigid, yet flexible stromata with colorations varying from deep to pale depending on the species. They also possess superficial, semi-immersed, or immersed perithecia, which are often obliquely situated and orderly arranged, and asci with conspicuous apical caps (Sung et al. 2007a).

As the largest genus within *Ophiocordycipitaceae* (Araújo et al. 2018; Luangsa-ard et al. 2018), *Ophiocordyceps* is distributed worldwide, with the highest species diversity occurring in tropical and subtropical regions (Petch 1933; Tzean et al. 1997; Ban et al. 2015). In China, the genus is also widely represented, with more than 30 new species described in recent years (Chen et al. 2021; Zou et al. 2022; Dai et al. 2024; Fan et al. 2024; Sun et al. 2024; Xu et al. 2025). Most species are insect pathogens, infecting a broad range of insect orders including, *Blattaria*, *Coleoptera*, *Diptera*, *Hemiptera*, *Hymenoptera*, and *Lepidoptera* (Evans et al. 2011; Luangsa-ard et al. 2018; Araújo and Hughes 2019). While some species have narrow host ranges, others, such as the *O. unilateralis* species complex, parasitize several genera of ants and exhibit a broader host spectrum, with certain species often able to infect multiple closely related host species (De Bekker et al. 2014; Kobmoo et al. 2019).

Asexual forms of *Ophiocordyceps* include *Hirsutella* Pat. (1892), *Hymenostilbe*Petch (1931), and *Paraisaria* Samson & B.L. Brady, (1983). Among these, *Hirsutella*-like and *Hymenostilbe*-like morphs are the most commonly observed (Sung et al. 2007a; Quandt et al. 2014; Araújo et al. 2018; Luangsa-ard et al. 2018; Khonsanit et al. 2019). These two asexual types are thought to have evolved independently within the genus (Luangsa-ard et al. 2011). *Hirsutella* is characterized by basally swollen or subulate phialides that taper to a point, with a mucilaginous packet containing one or several conidia at the apex (Simmons et al. 2015). *Hymenostilbe*, typified by *Hym. muscaria* Petch, produces immersed, oblique perithecia with filiform ascospores that dissociate into part-spores, with brightly colored, fleshy stromata (Petch 1931; Khonsanit et al. 2019). Its asexual morphs have cylindrical synnemata covered by a hymenium-like layer of conidiogenous cells. The phialides often have multi-toothed or wart-like projections at their surface, where smooth-walled conidia are produced (Petch 1931). Hosts of the *Hymenostilbe* clade mainly include adults of *Hymenoptera*, *Coleoptera*, *Diptera*, *Hemiptera*, and *Odonata*, with ants being particularly predominant (Kobayasi 1939; Khonsanit et al. 2019). Among described species in this clade, 20 species are associated with hymenopteran hosts (Sung et al. 2007a; Long et al. 2021; Yang et al. 2021).

*Hymenoptera* (including sawflies, wasps, ants, and bees) is one of the four most species-rich insect orders, with over 153,000 described species and an estimated total diversity potentially reaching one million (Rasplus et al. 2010; Aguiar et al. 2013; Peters et al. 2017). As parasitoids, predators, and pollinators, hymenopteran insects play crucial roles in nearly all terrestrial ecosystems, contributing significantly to biological control and biodiversity conservation (Eveleigh et al. 2007; Tscharntke et al. 2007). Their high species richness, substantial biomass, and broad distribution make them ideal hosts for the diversification of *Ophiocordyceps* (Hu et al. 2024).

During surveys of invertebrate-pathogenic fungi in Yunnan Province, China, several specimens infecting *Hymenoptera* were collected. Through combined morphological examination and molecular phylogenetic analyses, two new species and one new record belonging to the *Hymenostilbe* clade of *Ophiocordyceps* are introduced.

## Materials and methods

Stromata emerging from beneath fallen leaves were collected from subtropical, wet monsoon evergreen broad-leaved forests in Pu’er City and the Honghe Hani and Yi Autonomous Prefecture, Yunnan Province, China. Specimens were photographed and documented in the field using a Canon 90D digital camera and were then transferred into sterilized 50 mL plastic centrifuge tubes. Subsequently, specimens were placed in an insulated container with ice packs for transportation to the laboratory and were stored at 4 °C. Voucher specimens were deposited in the Cryptogamic Herbarium of the Kunming Institute of Botany, Chinese Academy of Sciences (**KUN-HKAS**).

### Morphological observations

Hosts and fruiting bodies of the newly collected specimens were photographed and measured using a Canon 90D digital camera and an OLYMPUS SZ61 dissecting microscope. Sections of the fertile parts were mounted in lactophenol cotton blue and 5% KOH, and microscopic morphological characteristics were examined under an OLYMPUS BX53 compound microscope. Measurements were conducted on perithecia, asci, ascospores, and part-spores, with at least 20 measurements taken for each structure (*n* ≥ 20), at magnifications of 10×, 20×, 40×, or 100×.

### DNA extraction, PCR, and sequencing

Genomic DNA was extracted from the stromata using the Ezup Column Fungi Genomic DNA Extraction Kit (Sangon Biotech Co., Ltd., Shanghai, China), following the manufacturer’s protocol. Polymerase chain reaction (PCR) was performed to amplify five loci using the following primer pairs: nrSSU-CoF/nrSSU-CoR for nuclear ribosomal small subunit (nrSSU) (Wang et al. 2015); LR0R/LR5 for nuclear ribosomal large subunit (nrLSU) (Vilgalys and Hester 1990; Rehner and Samuels 1994); EF1α-EF/EF1α-ER for translation elongation factor 1-alpha (*tef*-1α); RPB1-5'F/RPB1-5'R for the largest subunit of RNA polymerase II (*rpb*1); and RPB2-5'F/RPB2-5'R for the second largest subunit of RNA polymerase II (*rpb*2) (Bischoff et al. 2006; Sung et al. 2007b). Amplification of nrSSU and nrLSU was carried out following the protocols described by Fan et al. (2024), respectively, while amplification of *tef*-1α, *rpb*1, and *rpb*2 followed the methods of Bischoff et al. (2006).

The PCR was performed in a total volume of 23.5 μL, containing 12.5 μL of 2× Taq Master Mix (including Taq DNA polymerase, dNTPs, MgCl_2_, and reaction buffer), 1 μL each of forward and reverse primers, 1.5 μL of DNA template, and 7.5 μL of sterile deionized water. PCR conditions and cycling protocol followed the method described by Fan et al. (2024). Sequencing was conducted by Tsingke Biotechnology Co., Ltd. (Beijing, China).

### Phylogenetic analyses

**Figure 1.**
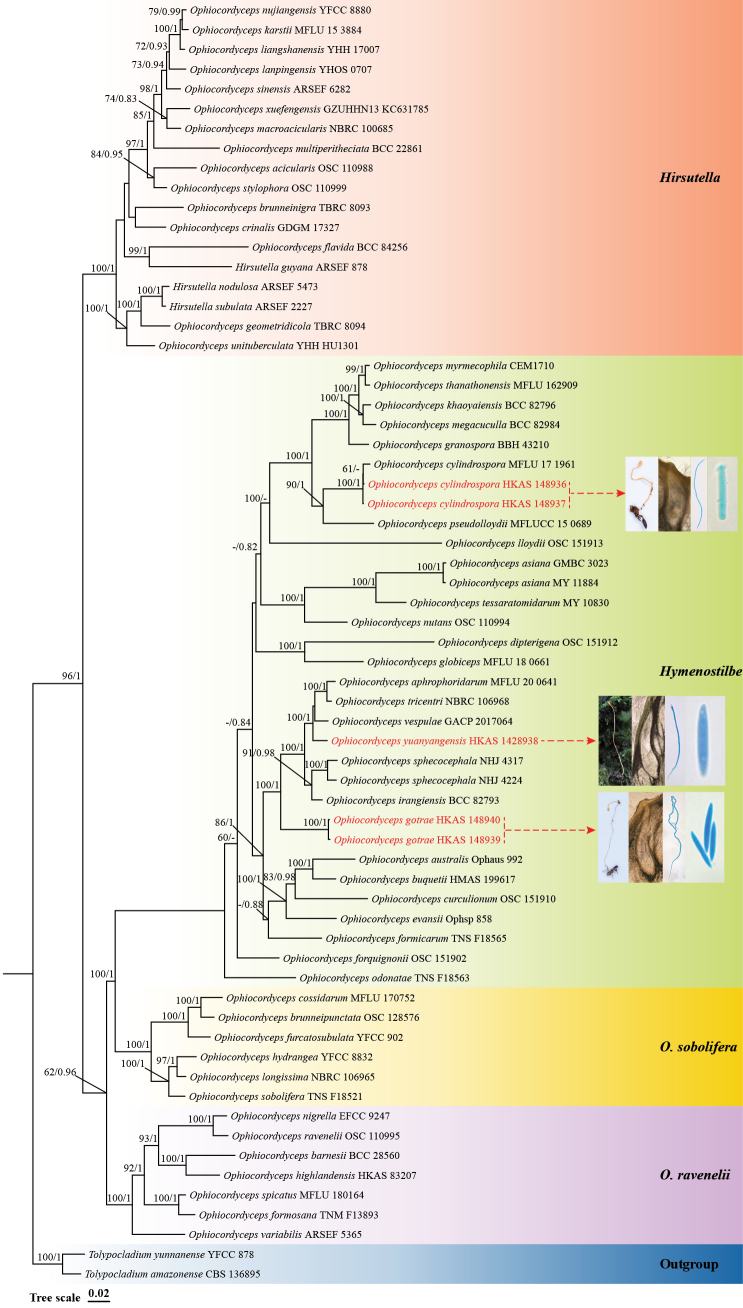
Phylogenetic tree based on the concatenated dataset of nrSSU, nrLSU, *tef*-1α, *rpb*1, and *rpb*2, showing the placements of two new species and a new Chinese record parasitic on *Hymenoptera* within *Ophiocordyceps*. Support values at the nodes before and after the backslash are the maximum likelihood (ML) bootstrap proportions (ML-BP > 70%) and Bayesian inference (BI) posterior probabilities (BI-PP > 0.60), respectively. Species described in this study are indicated in red.

Five loci (nrSSU, nrLSU, *tef*-1α, *rpb*1, and *rpb*2) were downloaded from NCBI (https://www.ncbi.nlm.nih.gov/) for sequences of *Hirsutella*, *Hymenostilbe*, *O. sobolifera* (Hill ex Watson) Sung et al., and *O. ravenelii* (Berk. & M.A. Curtis) Sung et al. clades within *Ophiocordyceps*. Five-gene sequences generated from five newly collected specimens were combined, resulting in a final dataset for phylogenetic analysis. *Tolypocladium yunnanense* Hong Yu et al. and *T. amazonense* Gazis, Skaltsas & P. Chaverri were selected as outgroups for phylogenetic tree construction. As members of the family *Ophiocordycipitaceae*, these species are phylogenetically closely related to *Ophiocordyceps*, ensuring robust rooting for the analysis. Specimen information and GenBank accession numbers for the five genes are provided in Table 1. Sequences were aligned using Clustal X v.2.0 and MEGA 6 software (Larkin et al. 2007; Tamura et al. 2013). A concatenated dataset comprising five loci—nrSSU, nrLSU, *tef*-1α, *rpb*1, and *rpb*2—was used. Phylogenetic trees were constructed using both Bayesian inference (BI) and maximum likelihood (ML) methods implemented in MrBayes v.3.1.2 and RAxML v.7.0.3, respectively (Ronquist and Huelsenbeck 2003; Stamatakis 2006). The optimal partitioning model (Table 2) was determined using PhyloSuite v.1.2.3 (Xiang et al. 2023). The generation number for the BI analysis was set at 5 million, while the ML analysis involved 1000 fast bootstrap replications. The resulting phylogenetic tree of *Ophiocordyceps* is illustrated in Fig. 1.

**Table 1.** Specimen information and GenBank accession numbers of sequences used in this study.

Species	Voucher information	Genbank accession number					Reference
		nrSSU	nrLSU	*tef*-1α	*rpb*1	*rpb*2	
*Hirsutella guyana*	ARSEF 878	KM652068	KM652111	KM651994	KM652035		Simmons et al. 2015
*H. nodulosa*	ARSEF 5473	KM652074	KM652117	KM652000	KM652040		Simmons et al. 2015
*H. subulata*	ARSEF 2227	KM652086	KM652130	KM652013	KM652051		Simmons et al. 2015
*Ophiocordyceps acicularis*	OSC 110988	EF468951	EF468804	EF468745	EF468853		Sung et al. 2007b
*O. aphrophoridarum*	MFLU 20-0641^T^	MW139324	MW139330	MW160163	MW160167	MW160165	Yang et al. 2021
*O. asiana*	GMBC 3023	PP583068	PP577935	PP681118	PP681108	PP681113	Guan et al. 2025
*O. asiana*	MY 11884		MW280216	MW292449	MW296052		Khao-Ngam et al. 2021
*O. australis*	Ophaus 992	KC610785	KC610766	KC610731	KF658663		Sanjuan et al. 2015
*O. barnesii*	BCC 28560	EU408776			EU408773	EU418599	Sanjuan et al. 2015
*O. brunneinigra*	TBRC 8093		MF614654	MF614638	MF614668	MF614681	Luangsa-ard et al. 2018
*O. brunneipunctata*	OSC 128576	DQ522542	DQ518756	DQ522324	DQ522369	DQ522420	Spatafora et al. 2007
*O. buquetii*	HMAS 199617	KJ878940	KJ878905	KJ878985	KJ879020		Quandt et al. 2014
*O. cossidarum*	MFLU 17-0752^T^	MF398186	MF398187	MF928403			Hyde et al. 2017
*O. crinalis*	GDGM 17327	KF226253	KF226254	KF226256	KF226255		Wang et al. 2014
*O. curculionum*	OSC 151910	KJ878918	KJ878885		KJ878999		Quandt et al. 2014
*O. cylindrospora*	MFLU 17-1961^T^	MG553651	MG553652			MG647029	Hyde et al. 2018
***O. cylindrospora***	**HKAS 148936**	**PV849292**	**PV849290**	**PV855903**	**PX406245**		**This study**
***O. cylindrospora***	**HKAS 148937**	**PV849293**	**PV849291**	**PV855904**	**PX406246**		**This study**
*O. dipterigena*	OSC 151912	KJ878920	KJ878887	KJ878967	KJ879001		Quandt et al. 2014
*O. evansii*	Ophsp 858	KC610796	KC610770	KC610736	KP212916		Sanjuan et al. 2015
*O. flavida*	BCC 84256		MT512655	MT533482	MT533476		Mongkolsamrit et al. 2021
*O. formicarum*	TNS F 18565	KJ878921	KJ878888	KJ878968	KJ879002	KJ878946	Quandt et al. 2014
*O. formosana*	TNM F13893	KJ878908		KJ878956	KJ878988	KJ878943	Quandt et al. 2014
*O. forquignonii*	OSC 151902	KJ878912	KJ878876		KJ878991	KJ878945	Quandt et al. 2014
*O. furcatosubulata*	YFCC 902^T^	MT774214	MT774221	MT774242	MT774228	MT774235	Wang et al. 2021
*O. geometridicola*	TBRC 8094		MF614647	MF614631	MF614664	MF614678	Luangsa-ard et al. 2018
*O. globiceps*	MFLU 18-0661	MH725812	MH725830	MH727388			Xiao et al. 2019
***O. gotrae***	**HKAS 148940^T^**	**PV865600**	**PV865601**	**PV855902**	**PV855906**		**This study**
***O. gotrae***	**HKAS 148939**	**PV865599**		**PV855901**	**PV855905**		**This study**
*O. granospora*	BCC 82256		MH028157		MH028169	MH028178	Khonsanit et al. 2019
*O. highlandensis*	HKAS 83207^T^	KM581284			KM581276	KM581280	Yang et al. 2015
*O. hydrangea*	YFCC 8832	OM304636	OM304640	OM831277	OM831280	OM831283	Zou et al. 2022
*O. irangiensis*	BCC 82793			MH028185	MH028163	MH028173	Khonsanit et al. 2019
*O. karstii*	MFLU 15 3884^T^	KU854952		KU854945	KU854943		Li et al. 2016
*O. khaoyaiensis*	BCC 82796^T^		MH028153	MH028187	MH028165	MH028175	Khonsanit et al. 2019
*O. lanpingensis*	YHOS 0707^T^	KC417459	KC417461	KC417463	KC417465		Chen et al. 2013
*O. liangshanensis*	YHH 17007	MT774220	MT774227	MT774248	MT774234	MT774241	Wang et al. 2021
*O. lloydii*	OSC 151913	KJ878924	KJ878891	KJ878970	KJ879004	KJ878948	Quandt et al. 2014
*O. longissima*	NBRC 106965	AB968392	AB968420	AB968584		AB968546	Ban et al. 2015
*O. macroacicularis*	NBRC 100685^T^	AB968388	AB968416	AB968574		AB968536	Ban et al. 2015
*O. megacuculla*	BCC 82984^T^		MH028162	MH028192		MH028181	Khonsanit et al. 2019
*O. multiperitheciata*	BCC 22861		MF614656	MF614640	MF614670	MF614683	Luangsa-ard et al. 2018
*O. myrmecophila*	CEM 1710		KJ878894	KJ878974	KJ879008		Quandt et al. 2014
*O. nigrella*	EFCC 9247	EF468963	EF468818	EF468758	EF468866	EF468920	Sung et al. 2007a
*O. nujiangensis*	YFCC 8880	ON723384	ON723381	ON868820	ON868823	ON868826	Sun et al. 2022
*O. nutans*	OSC 110994	DQ522549	DQ518763	DQ522333	DQ522378		Spatafora et al. 2007
*O. odonatae*	TNS F 18563		KJ878877		KJ878992		Quandt et al. 2014
*O. pseudolloydii*	MFLUCC 15 0689			MF372758	MF372761		Xiao et al. 2017
*O. ravenelii*	OSC 110995	DQ522550	DQ518764	DQ522334	DQ522379	DQ522430	Spatafora et al. 2007
*O. sinensis*	ARSEF 6282	KM652083	KM652126	KM652009	KM652048		Simmons et al. 2015
*O. sobolifera*	TNS F18521	KJ878933	KJ878898	KJ878979	KJ879013		Quandt et al. 2014
*O. sphecocephala*	NHJ 4317			GU797130			Luangsa-ard et al. 2011
*O. sphecocephala*	NHJ 4224			GU797131			Luangsa-ard et al. 2011
*O. spicatus*	MFLU 18-0164	MK863047	MK863054	MK860192			Zha et al. 2021
*O. stylophora*	OSC 110999	EF468982	EF468837		EF468882	EF468931	Sung et al. 2007a
*O. tessaratomidarum*	MY 10830^T^		MW280218	MW292434			Khao-ngam et al. 2021
*O. thanathonensis*	MFLU 16-2909		MF850377	MF872613	MF872615		Xiao et al. 2017
*O. tricentri*	NBRC 106968	AB968393	AB968423	AB968593		AB968554	Ban et al. 2015
*O. unituberculata*	YHH HU 1301^T^	KY923213		KY923215	KY923217	KY923219	Wang et al. 2018
*O. variabilis*	ARSEF 5365	DQ522555	DQ518769	DQ522340	DQ522386	DQ522437	Sung et al. 2007a
*O. vespulae*	GACP 2017064		MN044858	MN117075		MN117073	Long et al. 2021
*O. xuefengensis*	GZUHHN13	KC631785		KC631790	KC631795		Wen et al. 2013
***O. yuanyangensis***	**HKAS 148938^T^**	**PV865603**	**PV865602**	**PX516430**	**PX516429**		**This study**
*Tolypocladium amazonense*	CBS 136895	KF747314	KF747134	KF747099	KF747214		Gazis et al. 2014
*T. yunnanense*	YFCC 878	OP207720	OP207740	OP223154	OP223132		Dong et al. 2022

**Table 2.** Results of the best-fitting model for maximum likelihood (ML) and Bayesian inference (BI) for the five loci.

Gene name	Sites (bp)	ML (AIC)	BI (BIC)
nrSSU	804	TIM3e+I+I+R3	SYM+I+G4
nrLSU	1014	TIM+F+R4	GTR+F+I+G4
*tef*-1*α*	829	TIM2+F+I+I+R5	GTR+F+I+G4
*rpb*1	550	TIM+F+I+I+R4	GTR+F+I+G4
*rpb*2	859	TIM3+F+I+I+R7	GTR+F+I+G4

## Results

### Phylogenetic analyses

The phylogenetic analyses were conducted based on a concatenated five-gene dataset comprising 65 samples. The final aligned concatenated matrix had a total length of 4,156 bp, with the following partitions: nrSSU (903 bp), nrLSU (815 bp), *tef*-1α (880 bp), *rpb*1 (664 bp), and *rpb*2 (894 bp). Phylogenetic trees inferred from maximum likelihood (ML) and Bayesian inference (BI) analyses generated nearly consistent overall topologies, including four statistically well-supported clades within *Ophiocordyceps*: the *Hirsutella*, *Hymenostilbe*, *O. sobolifera*, and *O. ravenelii* clades (Fig. 1). This phylogenetic framework was consistent with previous studies (Simmons et al. 2015; Fan et al. 2024).

All five newly collected specimens (HKAS 148936, HKAS 148937, HKAS 148938, HKAS 148939, and HKAS 148940) from Yunnan, China, were placed in the *Hymenostilbe* clade with strong statistical support (ML/BI = 100/1). Among these, HKAS 148940 and HKAS 148939 were newly described as *O. gotrae*, and HKAS 148938 was newly described as *O. yuanyangensis*. These three specimens clustered together with *O. tricentri* (Yasuda) G.H. Sung et al., *O. aphrophoridarum* Yu Yang et al., *O. irangiensis* (Moureau) G.H. Sung et al., *O. sphecocephala* (Klotzsch ex Berk.) G.H. Sung et al., and *O. vespulae* F.Y. Long et al. within a strongly supported monophyletic clade in *Ophiocordyceps* (ML/BI = 100/1). The remaining two specimens, HKAS 148936 and HKAS 148937, clustered with *O. cylindrospora* (MFLU 17-1961), a species described from Thailand (Hyde et al. 2018), also with strong statistical support (ML/BI = 100/1). Thus, the phylogenetic results supported the recognition of *O. gotrae* and *O. yuanyangensis* as new species and *O. cylindrospora* as a new record of *Ophiocordyceps* in China.

### Taxonomy

#### Ophiocordyceps gotrae

Taxon classificationFungiHypocrealesOphiocordycipitaceae

Y.B. Wang, Z.M. Chen, H.F. Liao & Zhu L. Yang sp. nov.

B02BFC17-79C4-5BA3-AA28-9B1CFEB33AA8

Fungal Names: FN 573143

Fig. 2

##### Etymology.

Named after the host genus *Gotra* (*Hymenoptera*, *Ichneumonidae*), from which the holotype was collected as a parasite of *Gotra octocinctus*.

**Figure 2.**
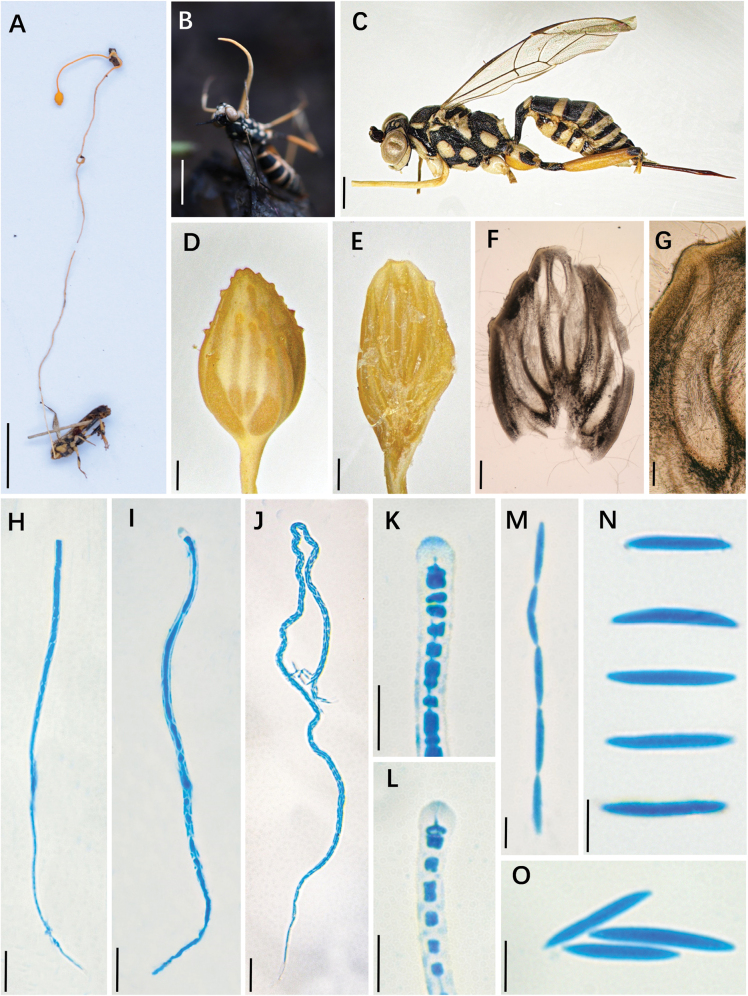
*Ophiocordyceps gotrae* (HKAS 148940). **A, B**. Fungus on host; **C**. Close-up of the host; **D**. Close-up of fertile part; **E, F**. Vertical sections of fertile part showing immersed perithecia; **G**. Perithecium; **H–J**. Asci; **K, L**. Apical caps; **M**. Part of ascospore; **N, O**. Part-spores. Scale bars: 10 mm (**A**); 5 mm (**B**); 1 mm (**C**); 200 μm (**D–F**); 100 μm (**G**); 20 μm (**H–J**); 10 μm (**K, L**); 5 μm (**M–O**).

##### Typus.

China • Yunnan Province, Honghe Hani and Yi Autonomous Prefecture, Luchun County, Huanglian Mountain, at 22°53'7.01"N, 102°16'16.23"E, alt. 1306 m, On an adult of *Gotra octocinctus* (*Hymenoptera*) emerging from the leaf litter on the forest floor, 11 August 2024, H.F. Liao (**holotype**HKAS 148940).

##### Description.

**Sexual morph: *Stromata*** solitary, arising from the lateral side of host thorax, pale yellow to yellow, 65–70 × 0.2–0.4 mm. ***Stipes*** wiry to pliant, pale yellow. ***Fertile parts*** terminal, elongated ellipsoid, pale yellow to yellow, 1.5–2.0 × 1.0–1.5 mm; surface spinous and distinctly textured, with protruding ostioles concentrated on the upper portion. ***Perithecia*** obliquely immersed, elongated obpyriform to narrowly flask-shaped, pale yellow to yellow, 901–1381 × 144–168 μm. ***Asci*** cylindrical, 200–300 × 2.2–3.8 μm. ***Apical cap*** hemigloboid to subglobose, with a central septum, 5.2–8.4 × 3.8–5.0 μm. ***Ascospores*** parallel arrangement, filiform, disarticulating into ***Part-spores*** of 11.7–13.3 × 1.4–2.1 μm, smooth, fusiform, gradually tapering toward both ends. **Asexual morph**: Undetermined. Attempts to isolate cultures were unsuccessful, and no asexual structures were observed on the specimens.

##### Host.

Adults of *Gotra octocinctus* (*Hymenoptera*).

##### Habitat.

In the leaf litter of wet monsoon evergreen broad-leaved forests, with bright stromata emerging from the dead leaf layer.

##### Geographic distribution.

China (Yunnan Province).

##### Additional materials examined.

China • Yunnan Province, Pu’er City, Zhenyuan Yi, Hani, and Lahu Autonomous County, Zhedong Town, at 23°57'29.37"N, 101°18'31.86"E, alt. 993 m, on an adult *Gotra octocinctus* (*Hymenoptera*), in leaf litter, 19 July 2024, H.F. Liao ( HKAS 148939).

##### Notes.

*Ophiocordyceps gotrae* is characterized by solitary, pale yellow to yellow stromata arising from the adult of *Gotra octocinctus* (*Hymenoptera*), bearing relatively small fertile parts at the apex, obliquely immersed perithecia that are elongated obpyriform to narrowly flask-shaped, short asci, and filiform ascospores disarticulating into fusiform part-spores.

Multi-gene phylogenetic analyses place *O. gotrae* in a distinct evolutionary clade together with *O. irangiensis* and *O. sphecocephala*, with strong statistical support (ML/BI = 100/1) (Fig. 1). These species share similar morphological traits, such as solitary yellow stromata and obliquely immersed perithecia (Hywel-Jones 1995, 1996). In addition, *O. gotrae* has smaller fertile parts compared to its close relatives (Table 3). They differ in host specificity: *O. gotrae* parasitizes *Gotra octocinctus*, whereas *O. irangiensis* parasitizes ants and *O. sphecocephala* parasitizes wasps (Hywel-Jones 1995, 1996). The morphological and molecular phylogenetic evidence robustly supports the recognition of *O. gotrae* as a new species within *Ophiocordyceps*.

**Table 3.** Morphological comparison of *Ophiocordyceps* species closely related to those in this study based on phylogenetic analysis.

Species	Distribution	Host	Stromata (mm)	Fertile parts (mm)	Perithecia (μm)	Asci (μm)	Ascospore (μm)	Part-spore (μm)	Reference
*O. aphrophoridarum*	China	*Aphrophoridae* sp.	Solitary, yellow, unbranched, 80–100 × 0.5–1.2	Cylindrical to fusiform, 10–20 × 2–5	Obliquely buried, ovoid to elongated pyriform, 638–798 × 108–178	filiform, 8-spored, hyaline, 337–445 × 6.1–8.7		Fusoid, 1-celled, straight, hyaline, 6.4-8.8 × 1.4–2.4	Yang et al. 2021
*O. cylindrospora*	Thailand	Wasp	Mostly single, pale yellow to yellow, 50–90 × 0.5–1.5	Fusiform, yellow, 3–3.2 × 1–1.2	Oblique, flask-shaped,551–638 × 261–327	Cylindrical,248–313 × 5–7	As long as asci	Cylindrical, 3.1–3.9 × 1.6–2	Hyde et al. 2018
*O. cylindrospora*	China	*Parapolybia varia* (*Hymenoptera*)	Solitary, pale yellow to yellow, 51–80 × 0.4–1.0	Elongated ellipsoid, surface roughened, 2.8–3.8 × 1.4–1.7	Obliquely immersed, ovoid to flask-shaped, 650.0–902.0 × 181.0–334.0	Cylindrical 360–622 × 2.2–6.3		Cylindrical, hyaline, smooth, flexural, 5.9–11.7 × 0.6–1.3	This study
*O. gotrae*	China	*Gotra octocinctus* (*Hymenoptera*)	Solitary, pale yellow to yellow, 65–70 × 0.2–0.4	Elongated ellipsoid, surface spinous,1.5–2.0 × 1.0–1.5	Obliquely immersed, elongated obpyriform to thin flask-shaped, 901.0–1381.0 × 144.0–168.0	Cylindrical, 200–300 × 2.2–3.8	Filiform hyaline	Fusiform, hyaline, smooth, 11.7–13.3 × 1.4–2.1	This study
*O. irangiensis*	Thailand	Ant	Usually single, yellow or orange, 25–60 × 0.2–0.7	Ovoid to sub-cylindric, 2.8-5.7 × 1.2-1.8	Immersed obliquely, elongated flask-shaped, 1000 × 150–200	Cylindric, capitate, 8-spored, 900 × 6–8	Filiform, multiseptate, 700 × 1.3-2.3	Fusoid, hyaline, 8.5–12.5 × 1.3–2.3	Hywel-Jones 1996
*O. sphecocephala*	Thailand	Wasp	Single, yellow, from head and thorax, 45 × 0.8–1.0	Citriform to cylindric, cream-yellow, 4–8 × 1.4–1.8	Immersed, obliquely vertical, elongated flask-shaped or conoid, 880–1000 × 200–260	Filiform, hyaline, 700 × 7	As long as asci	Hyaline, fusoid, 10–14 × 1.5–2.5	Hywel-Jones 1995
*O. tricentri*	Nepal	spittlebug (*Hemiptera*)	Solitary, yellow, 50–60 × 1–1.5	Ovoid, smooth, 5–6 × 1–1.5	Obliquely immersed, ovoid, 550–650 × 110–120	300–320 × 5			Shrestha and Sung 2005
*O. vespulae*	China	*Vespula* spp. (*Hymenoptera*)	Single or double, yellow, fibrous, cylindrical, 30–70 × 0.3–1	Cylindrical or elliptical, pale yellow, 5–10 × 0.5–1	Immersed, elongated flask-shaped, pale to yellowish, 520–720 × 200–380	Narrow cylindrical, hyaline, 320–570 × 5.3–7.5	Filiform, hyaline	Fusiform, hyaline, smooth, 7.5–11.5 × 1.5–3	Long et al. 2021
*O. yuanyangensis*	China	*Ichneumonidae (Hymenoptera)*	Solitary, pale yellow to yellow, 58.8 × 0.4	Fusiform or elongated ellipsoid, surface spinous, 3.8 × 1.2	Obliquely immersed, elongated obpyriform to thin flask-shaped, 1080.0–1130.0 × 164.0–202.0	Cylindrical, 208.0–270.0 × 2.7–4.8 μm	Filiform, 245.0–266.0 × 0.9–1.0	Hyaline, smooth, fusiform, 10.0–13.0 × 1.4–2.3 μm.	This study

#### Ophiocordyceps yuanyangensis

Taxon classification
Fungi
HypocrealesOphiocordycipitaceae

Y.B. Wang, H.F. Liao, Z.M. Chen & Zhu L. Yang sp. nov.

0D511729-BD79-5FEB-BA9E-840AB4927B5A

Fungal Names: FN 573144

Fig. 3

##### Etymology.

The specific epithet “*yuanyangensis*” refers to Yuanyang County, Yunnan Province, China, where the type specimen was collected.

##### Typus.

China • Yunnan Province, Honghe Hani and Yi Autonomous Prefecture, Yuanyang County, E’zha Township, at 22°59'5.4096"N, 102°27'31.8852"E, alt. 1882 m, on an adult of Ichneumonidae, emerging from the leaf litter on the forest floor, 21 July 2024, H.F. Liao (holotype HKAS 148938).

**Figure 3.**
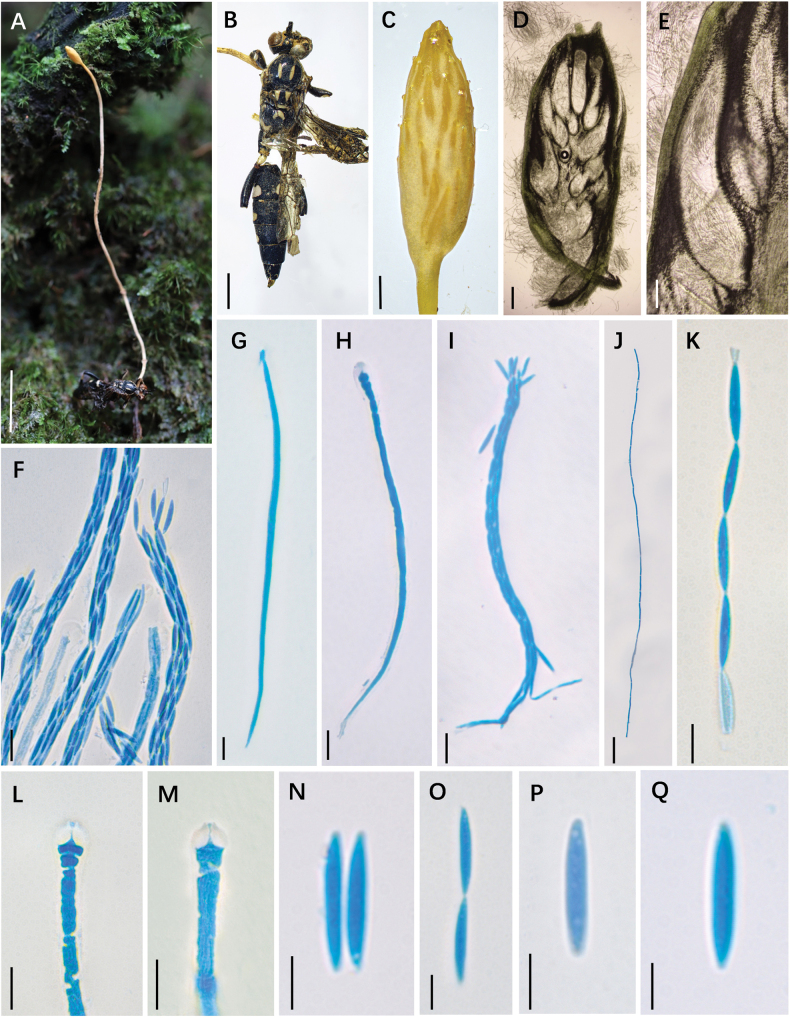
*Ophiocordyceps yuanyangensis* (HKAS 148938). **A**. Fungus on host; **B**. Close-up of the host; **C**. Close-up of fertile part; **D**. Vertical section of fertile part showing immersed perithecia; **E**. Perithecia; **F–I**. Asci; **J, K**. Ascospores; **L, M**. Ascus caps; **N–Q**. Part-spores. Scale bars: 10 mm (**A**); 500 μm (**B**); 500 μm (**C**); 200 μm (**D**); 100 μm (**E**); 10 μm (**F–I**); 20 μm (**J**); 5 μm (**K**); 10 μm (**L, M**); 5 μm (**N–Q**).

##### Description.

**Sexual morph: *Stroma*** solitary, arising from the anterior part of host thorax, pale yellow to yellow, 58.8 × 0.4 mm. ***Stipe*** wiry to pliant, pale yellow. ***Fertile part*** terminal, elongated ellipsoid or fusiform, surface spinous and distinctly textured, with protruding ostioles concentrated on the upper portion, pale yellow to yellow, 3.8 × 1.2 mm. ***Perithecia*** obliquely immersed, elongated obpyriform to narrowly flask-shaped, pale yellow to yellow, 1080–1130 × 164–202 μm. ***Asci*** cylindrical, 208–270 × 2.7–4.8 μm. ***Apical caps*** hyaline, hemigloboid, with a central septum, 7.1–8.2 × 4.9–5.6 μm. ***Ascospores*** parallel arrangement, filiform, 245–266 × 0.9–1.0 μm, disarticulating into part-spores at maturity. ***Part-spores*** smooth, fusiform, slightly tapering toward both ends, 10–13 × 1.4–2.3 μm. **Asexual morph**: Undetermined. Attempts to isolate cultures were unsuccessful, and no asexual structures were observed on the specimen.

##### Host.

Adult of Ichneumonidae (*Hymenoptera*).

##### Habitat.

In the leaf litter of subtropical broad-leaved evergreen forest, with bright stromata emerging from the dead leaf layer.

##### Geographic distribution.

China (Yunnan Province).

##### Notes.

*Ophiocordyceps yuanyangensis* is characterized by solitary, pale yellow to yellow stromata, obliquely immersed perithecia elongated obpyriform to narrowly flask-shaped, with protruding ostioles concentrated on the upper region of the fertile part, parallelly arranged, filiform ascospores, and fusiform part-spores.

Phylogenetically, *O. yuanyangensis* is closely related to *O. vespulae*, *O. tricentri*, and *O. aphrophoridarum*, forming a distinct clade with strong statistical support (ML/BI = 100/1) (Fig. 1). Pairwise sequence comparisons reveal clear nucleotide differences between the new species and these closely related taxa. Base-pair differences in the nrLSU and *tef*-1α sequences of *O. vespulae* are 2.12% (18/850) and 3.38% (31/918), respectively. Base-pair differences in the nrLSU, nrSSU, and *tef*-1α sequences of *O. tricentri* are 4.11% (35/851), 0.43% (4/941), and 4.36% (41/940), respectively. Base-pair differences in the nrLSU, nrSSU, *tef*-1α, and *rpb*1 sequences of *O. aphrophoridarum* are 4.12% (35/849), 0.53% (5/941), 4.42% (41/928), and 3.81% (27/708), respectively. Morphologically, *O. yuanyangensis* can be distinguished from related species by its relatively smaller fertile parts and asci, along with larger perithecia and part-spores (Long et al. 2021; Yang et al. 2021) (Table 3). In addition, they differ in host specificity: *O. yuanyangensis* parasitizes *Ichneumonidae (Hymenoptera)*, whereas *O. aphrophoridarum* parasitizes *Aphrophoridae (Hemiptera)*, *O. vespulae* parasitizes *Vespula* sp. (*Hymenoptera*), and *O. tricentri* parasitizes spittlebugs (*Hemiptera*) (Long et al. 2021; Yang et al. 2021) (Table 3).

#### Ophiocordyceps cylindrospora

Taxon classification
Fungi
HypocrealesOphiocordycipitaceae

Y.P. Xiao, T.C. Wen & K.D. Hyde

FCC312DC-03C8-57D9-B905-6D5580039E58

Index Fungorum: IF553983

Fig. 4

##### Typus.

Thailand • Chiang Mai Province, The Mushroom Research Center, on a dead wasp (*Hymenoptera*), 19 July 2015, Y.P. Xiao (**holotype**: MFLU 17-1961).

##### Description.

**Sexual morph: *Stromata*** solitary, arising from the dorsal thorax of insect hosts, pale yellow to yellow, 51–80 × 0.4–1.0 mm. ***Stipes*** wiry to pliant, yellow. ***Fertile parts*** terminal, elongated ellipsoid, yellow, surface roughened on the upper portion, ridged and furrowed laterally, with a coarse texture and prominent ostiolar openings, 2.8–3.8 × 1.4–1.7 mm. ***Perithecia*** obliquely immersed, ovoid to flask-shaped, yellow, 650–902 × 181–334 μm. ***Asci*** cylindrical, with a thickened apex, 360–622 × 2.2–6.3 μm. ***Apical caps*** hemiglobose to subglobose, with a small central septum, 7.6–9.1 × 5.6–7.8 μm. ***Part-spores*** smooth, flexuous, cylindrical, truncated at both ends, 5.9–11.7 × 0.6–1.3 μm. **Asexual morph**: Undetermined. Attempts to isolate cultures were unsuccessful, and no asexual structures were observed on the specimens.

**Figure 4.**
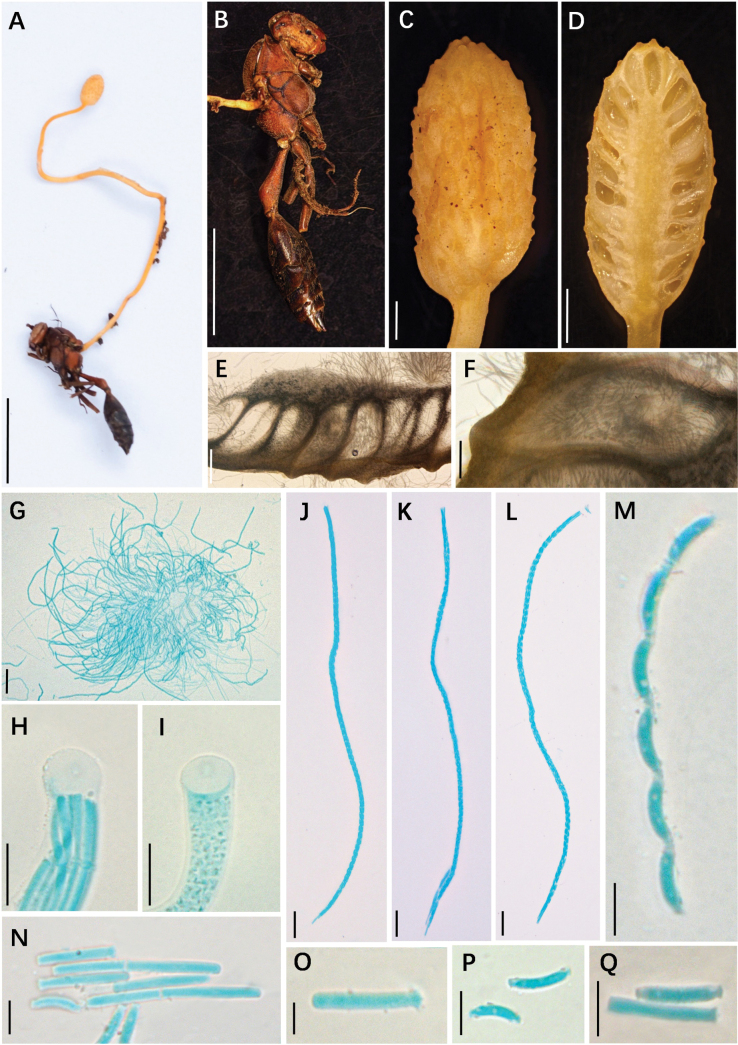
*Ophiocordyceps cylindrospora* (HKAS 148936). **A**. Fungus on host; **B**. Close-up of host; **C**. Close-up of fertile part; **D, E**. Vertical section of fertile part showing immersed perithecia; **F**. Perithecium; **G**. Asci; **H, I**. Apical caps; **J–L**. Asci; **M**. Part of ascospore; **N–Q**. Part-spores. Scale bars: 10 mm (**A**); 5 mm (**B**); 500 μm (**C, D**); 100 μm (**E–G**); 10 μm (**H, I**); 20 μm (**J–L**); 5 μm (**M–Q**).

##### Host.

Adults of *Parapolybia varia* (*Hymenoptera*).

##### Habitat.

In leaf litter of wet monsoon evergreen broad-leaved forests. Stromata emerge distinctly from the decaying leaf layer, usually during summer and autumn.

##### Geographic distribution.

China (Yunnan Province), Thailand (Chiang Mai Province).

##### Additional materials examined.

China • Yunnan Province, Honghe Hani and Yi Autonomous Prefecture, Luchun County, Huanglian Mountain, 22°53'7.01"N, 102°16'16.23"E, alt. 1306 m, on an adult *Parapolybia varia*, emerging from leaf litter on the forest floor, 11 Aug. 2024, Z.M. Chen (HKAS 148936). CHINA•Yunnan Province, Honghe Hani and Yi Autonomous Prefecture, Luchun County, 23°28'38"N, 102°24'12.68"E, alt. 1233 m, on an adult wasp, emerging from leaf litter on the forest floor, 18 August 2024, Z.M. Chen (HKAS 148937).

##### Notes.

*Ophiocordyceps cylindrospora* was first described from Thailand by Hyde et al. (2018). Its key diagnostic traits include solitary, pale yellow to yellow stromata, obliquely immersed, thin-walled, flask-shaped perithecia, narrow and cylindrical asci, cylindrical part-spores truncated at both ends, and parasitism on adult wasps (*Hymenoptera*).

Multi-gene phylogenetic analyses show that two specimens (HKAS 148936 and HKAS 148937) of *O. cylindrospora* collected in this study form a well-supported clade with the Thai material *O. cylindrospora* (MFLU 17-1961). Macroscopically, the two are highly similar; however, microscopic examination reveals differences in the size of the asci and part-spores (Table 3). In addition, the Thai material *O. cylindrospora* (MFLU 17-1961) parasitizes an unidentified wasp (Hyde et al. 2018), which is similar to *O. cylindrospora* (HKAS 148936), which parasitizes *Parapolybia varia* (*Hymenoptera*). Due to the macromorphological similarities between these hosts, it is inferred that they are phylogenetically close and belong to the same lineage. This study expands the known host range of this species and provides a more taxonomically refined identification of the host. These variations are consistent with intraspecific geographic divergence, supporting the identification of the Chinese specimen as *O. cylindrospora* while recognizing regional morphological plasticity.

## Discussion

Previous studies have shown that *Ophiocordyceps* species parasitizing *Hymenoptera* are primarily concentrated in two evolutionary lineages: the *O. unilateralis* subclade (also known as the *Hirsutella* ant pathogen subclade) within the *Hirsutella* clade and the *Hymenostilbe* clade (Simmons et al. 2015; Khao-Ngam et al. 2021; Xu et al. 2022; Tang et al. 2023). These two lineages are phylogenetically distant and exhibit markedly distinct morphological traits (Araújo et al. 2020; Tang et al. 2023). The *O. unilateralis* subclade, well known for causing the “zombie-ant” phenomenon, is characterized by fleshy, hemispherical fertile parts borne unilaterally on the stipe and is predominantly distributed in tropical rainforests (Evans et al. 2011; Tang et al. 2022). In contrast, species within the *Hymenostilbe* clade exhibit a broader host range but share common morphological features: they typically produce solitary, slender, pale yellow to orange, fleshy stromata, with terminally positioned fertile parts that often arise from gaps in the host’s head or thorax (Sung et al. 2007a; Khonsanit et al. 2019; Long et al. 2021). Microscopically, they possess immersed, obliquely oriented perithecia and filiform ascospores that disarticulate into 64 barrel- to column-shaped, flat-tipped part-spores (Khonsanit et al. 2019). These pronounced morphological differences provide strong support for their distant phylogenetic relationship.

In this study, *Ophiocordyceps* specimens parasitizing *Hymenoptera* were collected from Yunnan Province, China, and subjected to integrated morphological and multi-gene phylogenetic analyses. The results consistently indicated the presence of multiple lineages corresponding to new species. Accordingly, two new species, *O. yuanyangensis* and *O. gotrae*, are described, and the first Chinese record of *O. cylindrospora* is reported. Comparative morphological examination with closely related species, including *O. aphrophoridarum*, *O. tricentri*, and others (Table 3), revealed overall morphological similarity and diagnostic differences. For instance, *O. gotrae* possesses smaller fertile parts and shorter asci compared to its close relatives *O. sphecocephala* and *O. irangiensis*, whereas *O. yuanyangensis* differs from *O. aphrophoridarum*, *O. tricentri*, and *O. vespulae* in having smaller fertile parts and asci but larger perithecia and part-spores (Hywel-Jones 1995; Shrestha and Sung 2005; Hyde et al. 2018; Long et al. 2021; Yang et al. 2021). Although these species are morphologically similar and difficult to distinguish based solely on morphology, phylogenetic analyses provide robust support for their recognition as distinct species.

Currently, more than 40 species have been recorded in the *Hymenostilbe* clade. This clade comprises five primary groups: the *O. myrmecophila* complex, the *O. nutans* complex, the *O. sphecocephala* complex, the *O. dipterigena* complex, and the *O. australis* complex (Sung et al. 2007a; Khonsanit et al. 2019; Khao-Ngam et al. 2021; Dai et al. 2026). It also includes species parasitizing *Diptera*, such as the *O. dipterigena* complex (Berk. & Broome) G.H. Sung et al. and *O. globiceps* Y.P. Xiao, T.C. Wen & K.D. Hyde, as well as the *Odonata*-parasitizing species *O. odonatae* (Kobayasi) G.H. Sung et al. (Sung et al. 2007a; Xiao et al. 2019; Dai et al. 2026). Sanjuan et al. (2015) described *O. evansii*, belonging to the *O. australis* complex. Khonsanit et al. (2019) later conducted a comprehensive morphological and phylogenetic study of the *O. cf. myrmecophila* complex in Thailand, proposing three new species, *O. megacuculla*, *O. khaoyaiensis*, and *O. granospora*, and redefining lineages that previously lacked morphological data. All of these species share highly similar macromorphology and parasitize ants (*Hymenoptera*). Current consensus holds that host specificity represents a key diversification pattern for *Ophiocordyceps* species associated with ant hosts (Khonsanit et al. 2019). In 2021, the *O. nutans* complex was shown to comprise four cryptic species: *O. asiana* Mongkols. et al., *O. tessaratomidarum* Mongkols. et al., *O. nutans* (Pat.) G.H. Sung et al., and *O. neonutans* R. Friedrich et al. These species are widely distributed, share similar morphological characteristics, and parasitize adult stink bugs (*Hemiptera*, *Pentatomidae*) (Khao-Ngam et al. 2021). That same year, two species, *O. vespulae* F.Y. Long, Y.P. Xiao & T.C. Wen and *O. aphrophoridarum* Yu Yang, Y.P. Xiao & T.C. Wen, were described within the *O. sphecocephala* complex. This group exhibits a relatively broad host range, primarily infecting wasps but also including cicadas and ants. In summary, species within the *Hymenostilbe* clade are challenging to distinguish based on morphology alone due to overlapping macromorphological characteristics. Their host range spans multiple insect orders, predominantly *Hymenoptera* but also including *Diptera* and *Hemiptera*.

To date, 24 species within the *Hymenostilbe* clade are documented to parasitize *Hymenoptera*. These include *O. sphecocephala*, *O. vespulae*, *O. australis* (Speg.) G.H. Sung et al., *O. binata* (H.C. Evans & Samson) J.P.M. Araújo et al., *O. buquetii* (Mont. & C.P. Robin) Spatafora et al., *O. corriemoreauae* J.P.M. Araújo et al., *O. diabolica* J.P.M. Araújo et al., *O. evansii* Sanjuan, *O. granospora* Khons. et al., *O. irangiensis*, *O. oxycephala* (Penz. & Sacc.) G.H. Sung et al., *O. khaoyaiensis* Khons. et al., *O. lloydii* (H.S. Fawc.) G.H. Sung et al., *O. myrmecophila* (Ces.) G.H. Sung et al., *O. pseudomyrmicis* J.P.M. Araújo et al., *O. odontomachi* J.P.M. Araújo et al., *O. paltothyrei* J.P.M. Araújo et al., *O. pseudolloydii* (H.C. Evans & Samson) G.H. Sung et al., *O. thanathonensis* Y.P. Xiao et al., *O. megacuculla* Khons. et al., *O. longispora* (Samson & H.C. Evans) B. Shrestha et al., and *O. ponerus* X. Zou & Y.F. Han, as well as the two new species proposed here, *O. gotrae* and *O. yuanyangensis* (Hywel-Jones 1996; Sung et al. 2007a; Spatafora et al. 2015; Khonsanit et al. 2019; Qu et al. 2018; Araújo et al. 2020; Long et al. 2021). The primary hosts of these fungi are ants and wasps. However, morphological variation within the *O. sphecocephala* complex, *O. myrmecophila* complex, and *O. nutans* complex is minimal, and cryptic species are common, making accurate morphological identification challenging. Thus, molecular phylogenetic approaches are essential for delineating species boundaries. At the same time, broader sampling and formal species delimitation analyses will be needed in future studies to further assess species boundaries and potential hidden diversity (Khonsanit et al. 2019; Araújo et al. 2020; Khao-Ngam et al. 2021). The discovery of *O. yuanyangensis* and *O. gotrae* further expands the species diversity of this subclade and provides additional support for its phylogenetic classification.

### Medicinal importance

Among species in the *Hymenostilbe* clade that parasitize *Hymenoptera*, representative species such as *O. oxycephala*, *O. sphecocephala*, and *O. vespulae* have been documented to possess medicinal properties, including antitumor activity, reinforcement of deficiency, lung and kidney support, hemostasis, and phlegm resolution (Dai et al. 2009; Kornsakulkarn et al. 2018; Wu et al. 2019). Given that their hymenopteran hosts have substantial biomass in natural ecosystems (Rasplus et al. 2010; Peters et al. 2017) and that large aggregations of infected hosts are frequently observed in the field, other species within this clade likely possess similar medicinal potential. Therefore, continued research on this group holds considerable prospects for drug discovery. However, such investigations often face taxonomic challenges: many *Hymenoptera* species remain undescribed, and regional surveys are insufficient (Rasplus et al. 2010). This can complicate host identification and, in some cases, result in both the fungal pathogen and its insect host being new to science. Consequently, advancing research on entomopathogenic fungi must be accompanied by parallel efforts to accurately identify their insect hosts.

## Supplementary Material

XML Treatment for Ophiocordyceps gotrae

XML Treatment for Ophiocordyceps yuanyangensis

XML Treatment for Ophiocordyceps cylindrospora

## Additional information

### Artificial Intelligence (AI) use

The authors accept full responsibility for the content of the manuscript, including the disclosure of any use of AI.

No AI tools were used in the preparation of this manuscript.

### Author ORCIDs

Z.-M. Chen https://orcid.org/0009-0002-3010-433X

H.-F. Liao https://orcid.org/0009-0006-7287-1384

F.-W. Lou https://orcid.org/0009-0003-9065-7443

R. Hu https://orcid.org/0009-0004-2142-4686

J.-L. Jia https://orcid.org/0009-0009-2237-0989

W.-B. Zeng https://orcid.org/0009-0000-0251-5890

Z.-L. Yang https://orcid.org/0000-0001-9745-8453

A.-L. Zhang https://orcid.org/0000-0002-6169-6537

Y.-B. Wang https://orcid.org/0000-0002-3305-9418

### Data availability

All of the data that support the findings of this study are available in the main text or Supplementary Information.

## Supplementary materials

Supplementary material 1 A single gene tree Data type jpg https://binary.pensoft.net/file/1729671This dataset is made available under the Open Database License (http://opendatacommons.org/licenses/odbl/1.0/). The Open Database License (ODbL) is a license agreement intended to allow users to freely share, modify, and use this Dataset while maintaining this same freedom for others, provided that the original source and author(s) are credited. Ze-Mei Chen, He-Fa Liao, Fang-Wei Lou, Rui Hu, Jin-Long Jia, Wen-Bo Zeng, Zhu-Liang Yang, Ai-Li Zhang, Yuan-Bing Wang

Supplementary material 2 Alignment Data type fas https://binary.pensoft.net/file/1729672This dataset is made available under the Open Database License (http://opendatacommons.org/licenses/odbl/1.0/). The Open Database License (ODbL) is a license agreement intended to allow users to freely share, modify, and use this Dataset while maintaining this same freedom for others, provided that the original source and author(s) are credited. Ze-Mei Chen, He-Fa Liao, Fang-Wei Lou, Rui Hu, Jin-Long Jia, Wen-Bo Zeng, Zhu-Liang Yang, Ai-Li Zhang, Yuan-Bing Wang
